# Supplementary material for: Identification of photoperiod-induced specific miRNAs in the adrenal glands of Sunite sheep (Ovis aries)
Source: Front Vet Sci. 2022 Jul 22;9:888207. doi: 10.3389/fvets.2022.888207 (PMC9354845; doi:10.3389/fvets.2022.888207)
Supplement: Supplementary file 1 [file Data_Sheet_1.ZIP › Supplementary file/miRDeep2 file.zip!/miRDeep2 file/Know_Structure/LP42A2/oar-mir-376e.pdf]

miRBase precursor : oar-mir-376e
Total read count : 1261
oar-miR-376e-5p read count : 278
oar-miR-376e-3p read count : 705
oar-miR-376b-5p read count : 278
remaining reads : 0

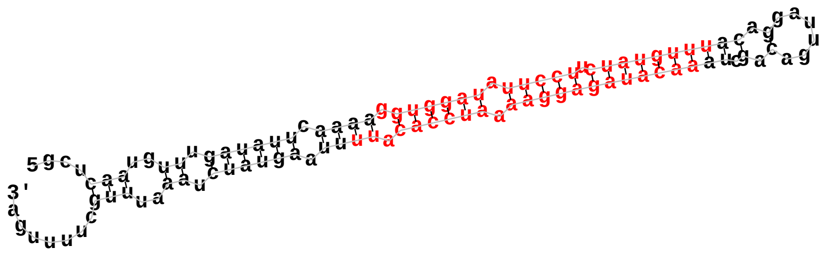

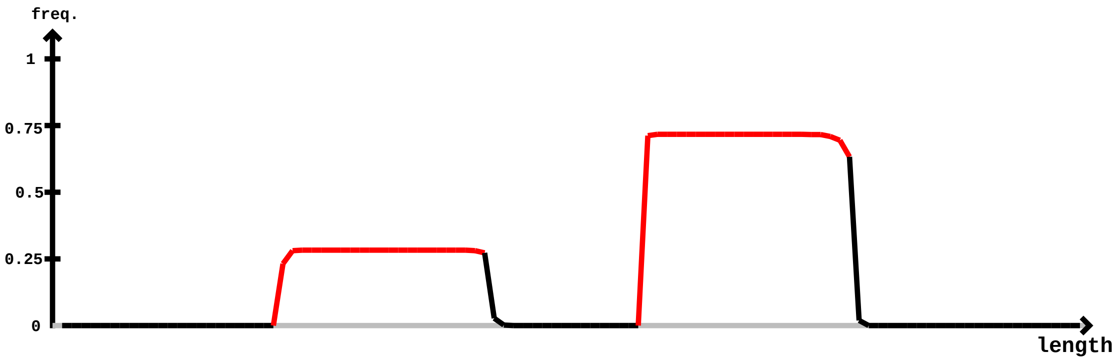


```
                                                            oar-miR-376e-3p
                      oar-miR-376b-5p
                      oar-miR-376e-5p
5'- gcucaauguuugauauucaaaagguggauauuccuucuauguuuacaggauugacagcuaaacauagaggaaaauccacauuuuaaguaucuaaauuugcuuuga   -3'    exp
    ((.....(((((((((.((((.((((((.(((((.((((((((((.(......).).))))))))))))))).)))))).)))).)))))).))))..))......   reads      mm        sample
    ......................gguggauauuccuucuaugu.....................................................................................   2      0        seq
    ......................gguggauauuccuucuauguu....................................................................................   7      0        seq
    ......................gguggauauuccuucuauguuu...................................................................................   200    0        seq
    ......................gguggauauuccuucuauguuua..................................................................................   19     0        seq
    .......................guggauauuccuucuauguu....................................................................................   1      0        seq
    .......................guggauauuccuucuauguuu...................................................................................   41     0        seq
    .......................guggauauuccuucuauguuua..................................................................................   6      0        seq
    ........................uggauauuccuucuauguuuac.................................................................................   2      0        seq
    ............................................................aacauagaggaaaaucA..................................................   1      1        seq
    ............................................................aacauagaggaaaauccac................................................   7      0        seq
    ............................................................aacauagaggaaaauccaca...............................................   14     0        seq
    ............................................................aacauagaggaaaauccacau..............................................   61     0        seq
    ............................................................aacauagaggaaaauccacauu.............................................   599    0        seq
    ............................................................aacauagaggaaaauccacauuu............................................   18     0        seq
    .............................................................acauagaggaaaauccacauu.............................................   4      0        seq
    .............................................................acauagaggaaaauccacauuu............................................   1      0        seq
```
